# Supplementary material for: A fault-intrusion-tolerant system and deadline-aware algorithm for scheduling scientific workflow in the cloud
Source: PeerJ Comput Sci. 2021 Nov 2;7:e747. doi: 10.7717/peerj-cs.747 (PMC8576554; doi:10.7717/peerj-cs.747)
Supplement: Supplemental Information 6 [file peerj-cs-07-747-s006.zip › Myproject1.1/docs/org/cloudbus/cloudsim/examples/network/datacenter/TestBagofTaskApp.html]

TestBagofTaskApp


JavaScript is disabled on your browser.


- Overview
- Package
- Class
- Use
- Tree
- Deprecated
- Index
- Help

- Prev Class
- Next Class

- Frames
- No Frames

- All Classes

- Summary:
- Nested |
- Field |
- Constr |
- Method

- Detail:
- Field |
- Constr |
- Method


org.cloudbus.cloudsim.examples.network.datacenter

## Class TestBagofTaskApp

- java.lang.Object
- - org.cloudbus.cloudsim.network.datacenter.AppCloudlet
  - - org.cloudbus.cloudsim.examples.network.datacenter.TestBagofTaskApp

- ---

    

  ```
  public class TestBagofTaskApp
  extends AppCloudlet
  ```

- - ### Field Summary

    - ### Fields inherited from class org.cloudbus.cloudsim.network.datacenter.AppCloudlet

      `accuracy, APP_MC, APP_Workflow, appID, clist, deadline, exeTime, numbervm, requestclass, type, userId`
  - ### Constructor Summary

    Constructors

    Constructor and Description

    `TestBagofTaskApp(int type,
    int appID,
    double deadline,
    int numbervm,
    int userId)`
  - ### Method Summary

    Methods

    | Modifier and Type | Method and Description |
    | --- | --- |
    | `void` | `createCloudletList(java.util.List<java.lang.Integer> vmIdList)` An example of creating APPcloudlet |
    | `int` | `getnumvm()` One can generate number of VMs for each application based on deadline |

    - ### Methods inherited from class java.lang.Object

      `clone, equals, finalize, getClass, hashCode, notify, notifyAll, toString, wait, wait, wait`

- - ### Constructor Detail


    - #### TestBagofTaskApp

      ```
      public TestBagofTaskApp(int type,
                      int appID,
                      double deadline,
                      int numbervm,
                      int userId)
      ```
  - ### Method Detail


    - #### createCloudletList

      ```
      public void createCloudletList(java.util.List<java.lang.Integer> vmIdList)
      ```

      **Description copied from class: `AppCloudlet`**

      An example of creating APPcloudlet

      **Overrides:**
      :   `createCloudletList` in class `AppCloudlet`

      Parameters:
      :   `vmIdList` - VMs where Cloudlet will be executed


    - #### getnumvm

      ```
      public int getnumvm()
      ```

      One can generate number of VMs for each application based on deadline

      Returns:


- Overview
- Package
- Class
- Use
- Tree
- Deprecated
- Index
- Help

- Prev Class
- Next Class

- Frames
- No Frames

- All Classes

- Summary:
- Nested |
- Field |
- Constr |
- Method

- Detail:
- Field |
- Constr |
- Method
